# Risk factors for acute asthma in tropical America: a case–control study in the City of Esmeraldas, Ecuador

**DOI:** 10.1111/pai.12401

**Published:** 2015-07-27

**Authors:** Cristina Ardura‐Garcia, Maritza Vaca, Gisela Oviedo, Carlos Sandoval, Lisa Workman, Alexander J. Schuyler, Matthew S. Perzanowski, Thomas A.E. Platts‐Mills, Philip J. Cooper

**Affiliations:** ^1^ Hospital ‘Delfina Torres de la Concha’EsmeraldasEsmeraldas ProvinceEcuador; ^2^ Laboratorio de Investigaciones FEPISQuinindéEsmeraldas ProvinceEcuador; ^3^ Clinical SciencesLiverpool School of Tropical MedicineLiverpoolUK; ^4^ Asthma and Allergic Diseases CenterUniversity of VirginiaCharlottesvilleVAUSA; ^5^ Department of Environmental Health SciencesMailman School of Public HealthColumbia UniversityNew YorkNYUSA; ^6^ Centro de Investigaciones en Enfermedades InfecciosasPontificia Universidad Católica del EcuadorQuitoEcuador; ^7^ Institute of Infection and ImmunitySt George's University of LondonLondonUK

**Keywords:** acute asthma, atopy, Ecuador, tropics, urban

## Abstract

**Background:**

Despite the high asthma rates described in Latin America, asthma risk factors in poor urban settings are not well established. We investigated risk factors for acute asthma among Ecuadorian children.

**Methods:**

A matched case–control study was carried out in a public hospital serving a coastal city. Children with acute asthma were age‐ and sex‐matched to non‐asthmatics. A questionnaire was administered, and blood, as well as stool, and nasopharyngeal swabs were collected.

**Results:**

Sixty cases and 119 controls aged 5–15 were evaluated. High proportions of cases were atopic with population‐attributable fractions for atopy of 68.5% for sIgE and 57.2% for SPT. Acute asthma risk increased with greater titers of mite IgE (3.51–50 kU/l vs. <0.70kU/l – OR 4.56, 95% CI 1.48–14.06, p = 0.008; >50kU/l vs. <0.70kU/l – OR 41.98, 95% CI: 8.97–196.39, p < 0.001). Asthma risk was significantly independently associated with bronchiolitis (adj. OR: 38.9, 95% CI 3.26–465), parental educational level (adj. OR 1.26, 95% CI: 1.08–1.46), and presence of sIgE (adj. OR: 36.7, 95% CI: 4.00–337), while a reduced risk was associated with current contact with pets (adj. OR: 0.07, 95% CI: 0.01–0.56). Rhinovirus infection was more frequent in cases (cases 35.6% vs. controls 7.8%, p = 0.002). None of the cases were on maintenance therapy with inhaled corticosteroids and most relied on emergency department for control.

**Conclusions:**

A high proportion of children presenting to a public hospital with acute asthma were allergic to mite, particularly at high IgE titer. Poor asthma control resulted in overuse of emergency care.

Asthma affects an estimated 300 million people worldwide 1 and is the most common chronic disease in children 2. Asthma is now as common in some Latin American countries as in known high prevalence countries such as the United Kingdom 3, mainly in poor urban settings 4, 5, where it is a significant public health problem 5. There are limited published data on asthma prevalence from poor populations in Latin American cities 1, 6. In Ecuador, 16% of the population 1 and 0.8–10.1% of rural schoolchildren 7, 8 have been estimated to have current asthma symptoms.

Asthma is a complex disease caused by interactions between environmental exposures and host genetics. Atopy, measured by skin test reactivity (SPT) or the presence of specific IgE (sIgE) to aeroallergens, is an important risk factor for asthma, but the population fraction of asthma attributable to atopy (PAF) varies greatly between regions 9.

The hygiene hypothesis has been put forward to explain temporal trends of increased asthma prevalence in industrialized countries 10 and a reduced prevalence among rural populations among whom exposure to farming and other factors associated with a more traditional rural lifestyle is considered to provide protection 11. It has been suggested that the hygiene hypothesis may not apply to urban Latin America 12 where prevalence rates are particularly high among the poor who live in unhygienic and overcrowded conditions 13.

Inadequate asthma management and control is frequent in Latin America 14, where asthmatic children often rely on emergency care for management of their disease 15. Acute asthma exacerbations are episodes of increasing shortness of breath, accompanied by cough, wheezing, and chest tightness, although there is disagreement on a precise definition 16. Severe acute asthma has been defined as that requiring systemic corticosteroids and emergency department attendance or hospital admission 17.

The aim of this study was to identify risk factors for asthma among children presenting with an acute exacerbation to the emergency department at the public hospital serving a small coastal city in Ecuador.

## Methods

### Study design and population

A matched case–control study was carried out between October and December 2012 at the public hospital, Hospital ‘Delfina Torres de Concha’ (HDTC) in the City of Esmeraldas in northeastern coastal Ecuador. Children aged 5–15 attending the emergency department at HDTC with a clinical presentation consistent with acute asthma (defined as respiratory distress and wheezing with or without coughing that improved on administration of bronchodilators) 17 were eligible as cases. Controls were children attending the emergency department for reasons other than asthma. Two controls were matched to each case by age (±2 years) and sex. Exclusion criteria were no parental consent and children who had other chronic respiratory (e.g., cystic fibrosis and pulmonary hypertension), cardiac (e.g., congenital heart disease) or neurological (e.g., cerebral palsy and Down syndrome) diseases.

### Study procedures

A questionnaire based on International Study of Asthma and Allergies in Childhood (ISAAC) phase II questionnaire 18 was administered in Spanish to the child's parent or guardian to collect information on socio‐demographic factors, core allergy and asthma symptoms, risk factors, and previous treatment and management of asthma. This questionnaire has been widely field‐tested in Ecuador 7, 19. Bronchiolitis was defined as an episode of wheeze and difficulty breathing during the first two years of life 20. Weight and height were measured. A blood sample was taken to measure hematocrit, total white cell and differential count, and total and specific IgE.

### IgE measurements

Total IgE and IgE antibodies (sIgE) specific for *Dermatophagoides pteronyssinus, D. farinae, Blomia tropicalis, Periplaneta americana* (American cockroach), cat, dog, grass, *Blatella germanica* (German cockroach), *Alternaria*,* Aspergillus*, and *Ascaris* were measured in plasma using ImmunoCAP (Phadia, Uppsala, Sweden) according to the manufacturer's instructions. A positive assay for sIgE was defined as >0.70 kU/l.

### Allergen skin prick test

Skin prick testing was carried out with eight allergen extracts (Greer laboratories, Lenoir, NC, USA): house dust mite (*Dermatophagoides pteronyssinus/D.farinae* mix), tropical mite (*Blomia tropicalis*, Leti, Spain), grass pollen, American cockroach, fungi, *Alternaria tenuis*, cat, dog, and positive histamine and negative saline controls. A positive reaction was defined as a mean wheal diameter ≥ 3 mm than the saline control 15 min after pricking the allergen onto the forearm. The test was undertaken at the moment of recruitment during the acute attack. Children taking antihistamines were excluded from SPT analyses.

### Stool examinations

Single stool samples were collected up to 2 weeks after recruitment and analyzed for geohelminth eggs and larvae by direct wet mount and formol‐ethyl acetate concentration methods 21.

### Airways function and inflammation

Forced expiratory volume in the first second and FVC were measured before and after administering a β_2_ agonist (2 puffs of Ventolin, GlaxoSmithKline, UK) using a portable spirometer (MicroMedical, UK). Fraction of exhaled nitric oxide (FeNO) was measured using an adapted NO breath instrument (Bedfont, UK) at flow rates of 50, 83, and 100 ml/sec, as described 22. These results were used to estimate FeNO parameters (alveolar concentration and bronchial flux) using Hogman method 23.

### PCR for respiratory viral infections

A nasopharyngeal swab was collected into universal transport medium (Diagnostic Hybrids, Athens, OH, USA) and stored at −80C. RNA was extracted using commercial kits (Qiagen), and specific RNA for rhinovirus, respiratory syncytial virus (RSV), human metapneumovirus (HMPV) and parainfluenza viruses (PIV) 1–4 was converted to cDNA and amplified by reverse transcriptase real‐time PCR (AgPath‐ID™ One‐Step RT‐PCR kit, Invitrogen) using primer/probe combinations as described 24 on a 7500 Fast machine (Applied Biosystems). A positive test was defined as Ct values ≤38.

### Statistical analysis

We estimated that 60 asthma cases and 120 non‐asthmatic controls would give 90% power at p < 0.05 to detect OR ≥2.2 or ≥1.8 for asthma risk factors with 10% or 30% prevalence, respectively. Fisher's exact test and Mann–Whitney U‐test were used to compare groups, as appropriate. The associations between acute asthma and risk factors were explored using multivariate conditional logistic regression. The population fraction of acute asthma attributable to atopy (PAF%) was estimated [(OR‐1/OR) × prevalence of atopy among cases]. Statistical analysis was carried out using spss Statistics version 20.0 (Armonk, New York, United States).

### Ethical Approval

The study protocol was approved by the Bioethics Committees of the Universidad San Francisco de Quito and Pontificia Universidad Católica del Ecuador, Quito, Ecuador. Informed written consent was obtained from the child's parent or guardian and minor's assent from the child.

## Results

A total of 179 children (60 cases and 119 matched controls) were recruited. Differences between cases and controls for study variables are shown in Table 1. Atopy was much more common in cases than controls and cases had higher levels of total IgE. Cases had higher levels of bronchial NO but not alveolar NO. Few of the children were infected with intestinal helminths: 4 had *Ascaris lumbricoides* and 3 had *Trichuris trichiura*. A history of bronchiolitis during infancy was associated with acute asthma and did not differ significantly by age and sex (Table S1).

**Table 1:** Characteristics of cases and controls with respect to study variables

Variable	Cases (N = 60)	Controls (N = 119)	p value
*Demographics*			
Age, median (IQR)	9 (6–11)	10 (7–12)	0.226
Sex, Male (%)	31 (52)	63 (53)	0.876
Ethnicity (%)			
Afro‐Ecuadorian	18 (30)	31 (26)	0.814
Mestizo or other^a^	42 (70)	86 (74)	
*Socioeconomic*			
Monthly income, median (IQR)	500 (224–800)	300 (200–500)	0.004
Cash transfer^b^ (%)	12 (20)	53 (45)	0.002
Parental school years^c^, Median (IQR)	26 (24–30)	21 (18–24)	<0.001
*Early childhood exposures*			
Birthweight (%)			
Low (<2.5 kg)	11 (26)	6 (9)	0.101
Normal	32 (74)	60 (91)	
Breastfed (%)	60 (100)	115 (98)	0.552
Contact with pets, 1st year (%)	33 (55)	63 (53)	0.874
Contact with farm animals, 1st year (%)	5 (8)	10 (9)	1.000
Air conditioning^d^, 1st year (%)	4 (7)	1 (1)	0.044
Carpeting, 1st year^d^ (%)	13 (22)	5 (4)	0.001
Humid household^e^, 1st year (%)	19 (32)	38 (33)	1.000
Bronchiolitis (%)	38 (63)	16 (14)	<0.001
Day care attendance before 4 years	12 (20)	38 (32)	0.112
*Current exposures*			
Parental asthma (%)	27 (45)	28 (24)	0.006
Birth order, median (IQR)	2 (1–3)	2 (1–4)	0.114
Contact with pets (%)	42 (70)	109 (92)	<0.001
Contact with farm animals (%)	8 (13)	14 (12)	0.815
Household ETS	9 (15)	29 (24)	0.177
Humid house^e^ (%)	19 (32)	45 (38)	0.510
Air conditioning^d^ (%)	6 (10)	5 (4)	0.185
Carpeting^d^ (%)	8 (13)	12 (10)	0.616
Intestinal helminths (%)	1 (2)	6 (8)	0.419
Rhinovirus infection (%)	21 (36)	8 (8)	<0.001
*Nutritional status*			
BMI, median (IQR)	16.1 (15.0–18.7)	16.9 (15.4–19.8)	0.184
Hematocrit, median (IQR)	40 (38–43)	40 (38–41)	0.125
*Allergic parameters*			
Eosinophil counts, median (IQR)	298 (128–515)	263 (93–506)	0.532
SPT, any allergen (%)	41 (68)	26 (22)	<0.001
asIgE, any allergen (%)	49 (82)	49 (43)	<0.001
Total IgE [kU/l], median (IQR)	837 (267–1264)	204 (67–672)	<0.001
*Airways function/ inflammation*			
FEV1/FVC (IQR)	0.83 (0.77–0.88)	0.88 (0.85–0.93)	<0.001
Reversibility with B2 agonist (%)	13 (28)	7 (10)	0.012
Alveolar NO (ppb), median (IQR)^f^	13.0 (10.0–21.6)	12.4 (7.7–17.9)	0.196
Bronchial NO flux (pl/sec), median (IQR) f	649 (300–1097)	317 (−16–652)	0.004

BMI, body mass index; IQR, interquartile range; ETS, exposure to tobacco smoke; SPT, skin prick test; asIgE, allergen‐specific IgE; FEV1, forced expiratory volume in the first second; FVC, forced vital capacity; NO, nitric oxide. Missing data: parental school years (10), monthly income (1), birthweight (70), breastfeeding (1), contact with farm animals 1st year (3), humidity in household 1st year (3), bronchiolitis (1), day care attendance (1), parental asthma (1), birth order (2), contact with farm animals (5), humid house (1), intestinal helminths (60), BMI (17), hematocrit/eosinophil counts (5), SPT (2), asIgE/total IgE (6), FEV1/FVC and reversibility (60), and alveolar NO/bronchial NO flux (57).

a Mestizos/whites; cases 41/1; controls 85/3.

b Beneficiary of conditional cash transfer program, ‘Bono Solidario’.

c Total number of years of school attendance by mother and father.

d Anywhere in the house.

e Damp spots anywhere in the house.

f FeNO parameters were estimated with the measurements at flow rates: 50, 83 and 100 ml/sec, using Hogman method (see Methods).

The results of multivariate analyses are provided in Table 2. Significant risk factors for acute asthma were bronchiolitis in early childhood, atopy (any positive sIgE), and higher parental educational level. Current contact with domestic pets was associated with reduced risk of asthma.

**Table 2:** Conditional logistic regression for risk factors for acute asthma

	Crude OR^a^	95% CI	p value	Adjusted OR^b^	95% CI	p value
Bronchiolitis	19.6	6.00–64.1	<0.001	38.9	3.26–465	0.004
Parental asthma	2.68	1.34–5.35	0.005	4.88	0.83–28.8	0.080
Atopy (sIgE)	6.06	2.65–13.9	<0.001	36.7	4.00–337	0.001
Carpeting 1st year	5.94	1.92–18.3	0.002	0.21	0.01–3.08	0.255
Air conditioning 1st year	8.00	0.89–71.6	0.063			
Current contact with pets	0.22	0.09–0.53	0.001	0.07	0.01–0.56	0.012
Parental school years	1.17	1.09–1.26	<0.001	1.26	1.08–1.46	0.003
Monthly income	1.00	1.00–1.00	0.064			

OR, odds ratio; CI, confidence interval. Atopy is defined as any asIgE>0.70 kU/l.

a Crude odds ratios were calculated by conditional logistic regression (matching variables – age and sex).

b Adjusted odds ratios were adjusted for all other variables in the model.

The associations between markers of atopy to specific aeroallergens and acute asthma are shown in Table 3. For SPT, we observed strong associations between reactivity to mites and asthma, and a weaker association with SPT to American cockroach. Few children were reactive to other aeroallergens: grass pollen (0% vs. 2%, p = 0.509), *Alternaria* (2% vs. 0%, p = 0.582), cat dander (2% vs. 4%, p < 0.001) and none for dog or fungi mix. The population fraction of acute asthma attributable to SPT to any aeroallergen was 57.2%.

**Table 3:** Associations between atopy and acute asthma

Atopic parameter	Cases	Controls	OR^a^ (95% CI)	p value
SPT (%)	N = 60	N = 117		
Any aeroallergen	41 (68)	26 (22)	6.31 (3.02–13.2)	<0.001
Any mite	41 (68)	20 (17)	9.94 (4.17–23.7)	<0.001
*Dermatophagoides pteronyssinus*/*farinae*	39 (65)	19 (16)	8.38 (3.70–19.0)	<0.001
*Blomia tropicalis*	14 (23)	5 (4)	8.10 (2.30–28.5)	0.001
American cockroach	12 (20)	10 (9)	2.68 (1.03–6.96)	0.043

Specific IgE (%, >0.7 kU/l)	N = 60	N = 113		
Any positive aeroallergen	49 (82)	49 (43)	6.06 (2.65–13.9)	<0.001
Any mite (%)				
≤0.7	11 (18)	70 (62)	1	
0.71–3.5	6 (10)	26 (23)	1.83 (0.55–6.06)	0.324
3.51–50	12 (20)	13 (12)	4.56 (1.48–14.1)	0.008
>50	31 (52)	4 (3)	42.0 (8.97–196)	<0.001
*Dermatophagoides pteronyssinus* (%)				
≤0.7	19 (32)	83 (73)	1	
0.71–3.5	3 (5)	21 (19)	0.73 (0.20–2.72)	0.638
3.51–50	17 (28)	7 (6)	8.77 (2.46–31.2)	0.001
>50	21 (35)	2 (2)	–	0.850
*Dermatophagoides farinae* (%)				
≤0.7	14 (23)	77 (68)	1	
0.71–3.5	6 (10)	20 (18)	1.57 (0.47–5.31)	0.465
3.51–50	23 (38)	15 (13)	6.66 (2.49–17.8)	<0.001
>50	17 (28)	1 (1)	–	0.858
*Blomia tropicalis* (%)				
≤0.7	13 (22)	76 (67)	1	
0.71–3.5	8 (13)	21 (19)	2.45 (0.82–7.35)	0.110
3.51–50	18 (30)	14 (12)	7.06 (2.41–20.7)	<0.001
>50	21 (35)	2 (2)	36.5 (7.63–175)	<0.001
American cockroach (%)^b^				
≤0.7	38 (63)	91 (81)	1	
0.71–3.5	12 (20)	15 (13)	1.92 (0.80–4.60)	0.143
>3.5	10 (17)	7 (6)	2.98 (1.10–8.03)	0.031
Ascaris (%)^b^				
≤0.7	26 (43)	75 (66)	1	
0.71–3.5	16 (27)	21 (19)	1.94 (0.91–4.10)	0.084
>3.5	18 (30)	17 (15)	2.59 (1.22–5.49)	0.013

OR, odds ratio; CI, confidence interval; SPT, skin prick test.

a Odds ratios were calculated by conditional logistic regression (adjusted matching variables: age and sex).

b No further stratification of IgE levels for American cockroach and Ascaris, given the small number of patients and therefore lack of power. The proportions were as follows: American cockroach: cases: 3.51–50 kU/l: 10 (17%), >50kU/l: 0; controls: 3.51–50 kU/l: 6 (5%), >50kU/l: 1 (1%). Ascaris: cases: 3.51–50 kU/l: 17 (28%), >50kU/l: 1 (2%); controls: 3.51–50 kU/l: 17 (15%), >50kU/l: 0.

**Figure 1:**
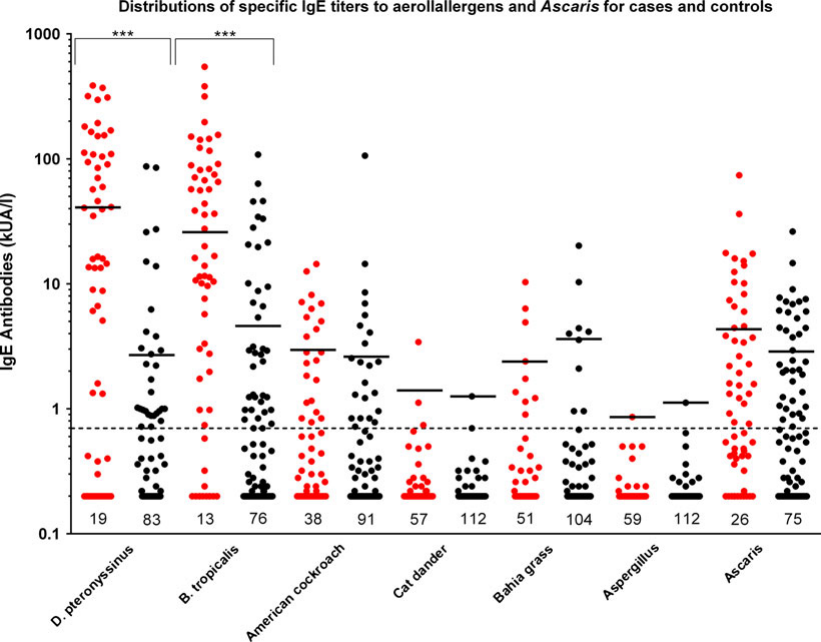
Distributions of IgE titers (kUA/l) among cases and controls for each aeroallergen and Ascaris. Threshold (horizontal dotted line) for a positive assay was 0.70 kUA/l. Numbers represent negatives. Red: cases and Black: controls. Geometric means of positive values (i.e., ≥0.70 kUA/l) are shown by horizontal lines and were for cases vs. controls, respectively – *D. pteronyssinus*, 41.2 vs. 2.69, p < 0.001; *B. tropicalis* 26.2 vs. 4.64, p < 0.001; American cockroach, 2.94 vs. 2.58, p = 0.411; cat, cases: 1.42 vs. 1.26, p = 1.000; grass, 2.36 vs. 3.61, p = 0.489; Aspergillus, 0.86 vs. 1.12, p = 1.000; Ascaris, 4.32 vs. 2.85, p = 0.149. Not shown: *D. farinae*, cases: 25.2 vs. 4.07, p < 0.001; German cockroach, 2.79 vs. 2.44, p = 0.650; dog, 2.32 vs. 1.11, p = 0.556; Alternaria, <0.70 vs. 0.92. ***: p < 0.001.

The data for sIgE are shown in Fig. 1 and Table 3. The population fraction of acute asthma attributable to specific IgE to any aeroallergen was 68%. We observed a strong association between sIgE to mite (any) and risk of acute asthma (cases 82% vs. controls 39%, OR 5.55, 95% CI: 2.54–12.1, p < 0.001), as well as for each of the mite allergens. The risk of acute asthma increased with increasing mite IgE titer (Table 3). The presence of IgE to American (OR 2.32, 95% CI: 1.14–4.71, p = 0.020) and German (OR 2.55, 95% CI: 1.23–5.27, p = 0.012) cockroach was also associated with asthma. The presence of IgE to other aeroallergens was not associated with acute asthma: cat (cases 5% vs. controls 1%, p = 0.121), dog dander (7% vs. 5%, p = 0.721), grass mix (15% vs. 8%, p = 0.191), *Alternaria* (0 vs. 1%, p = 1), and Aspergillus (2% vs. 1%, p = 1). Few children had high‐titer IgE (>3.5 kU/l) to other aeroallergens (Fig. 1) among whom there was limited evidence of an increasing risk with increasing titer. The presence of *Ascaris* IgE was associated with acute asthma (cases 57% vs. controls 34%; OR 2.24, 95% CI: 1.22–4.10, p = 0.009) and risk increased with increasing titer. Because mite and Ascaris IgE titers were strongly correlated (r = 0.748, p < 0.001), we explored whether the association between acute asthma and Ascaris IgE might be explained by mite IgE. After controlling for mite IgE, the association between asthma and Ascaris IgE was lost (Ascaris IgE, 0.71–3.5 vs. ≤0.7 kU/l – OR 0.43, 95% CI 0.13–1.41, p = 0.164; >3.5 vs. ≤0.7 – OR 0.39, 95% CI 0.11–1.38, p = 0.143), while the risk of acute asthma remained strongly positive for mite IgE (OR 1.05, 95% CI 1.02–1.07, p < 0.001). The presence of *Ascaris* IgE decreased the odds of a positive SPT to any aeroallergen (OR: 0.26, 95% CI: 0.13–0.49, p < 0.001). The population fractions of asthma attributable to mite IgE and *Ascaris* IgE were 64.3% and 31.4%, respectively.

We analyzed nasopharyngeal swabs from 59 cases and 103 controls: 35.6% of cases had evidence of rhinovirus infection compared to 7.8% of controls (OR 3.81, 95% CI 1.67–8.73, p = 0.002) but positivity for other viruses was not significantly different: RSV (0 vs. 4.9%, p = 0.160), PIV 1 (1.7% vs. 0%, p = 0.364), PIV 2 (1.7% vs. 3.9%, p = 0.654), and PIV 4 (6.8% v.s 5.8%, p = 1). No swabs were positive for HMPV or PIV 3.

We explored the effect of rhinovirus infection on the association between acute asthma and mite (any) IgE titer (Table S2). There was some evidence of a stronger association between mite IgE and acute asthma among children with rhinovirus infection compared to those without infection, but the small number of infections made the estimates of effect extremely imprecise.

**Figure 2:**
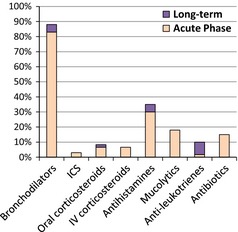
Treatment received during the previous 12 months by children with acute bronchospasm. ICS, inhaled corticosteroids; IV, intravenous.

Of the 60 acute asthma cases evaluated, 77% had been previously diagnosed with asthma, 20% had visited a doctor during the last year for regular asthma control, and 86% had attended the emergency department at least once in this same period with an acute exacerbation. Treatments received in the past year are shown in Fig. 2. Almost all children had used oral bronchodilators (91%) during acute attacks. Five children had been taking antileukotriene agents (montelukast) for long‐term treatment as monotherapy, and none were receiving inhaled corticosteroids.

## Discussion

In the present study, we have investigated risk factors for acute asthma among children aged 5 to 15 attending the emergency room of a public hospital in a small coastal city in tropical Ecuador. Our data show that the majority of children (82%) with acute bronchospasm in our population are atopic with a population fraction of acute asthma attributable to sIgE of 68.5%. Mites were by far the most important sensitizing aeroallergens. The odds for acute asthma increased with mite sIgE titer: such that at a titer of greater than 3.5 kU/l, the risk of acute asthma was approximately fivefold greater, and at titers greater than 50 kU/l, the odds of asthma were almost 40‐fold greater than controls.

The ISAAC phase II study estimated population‐attributable fractions (PAFs) of wheeze/asthma among children aged 8–12 attributable to sIgE of 18.3% in non‐affluent compared to 45.6% in affluent countries 9. The two Latin American study centers in ISAAC phase II estimated PAFs of 11% for wheeze/SPT 9. Previous estimates of PAFs in Esmeraldas Province in case–control studies of current wheeze/SPT have estimated 10.1% in rural and 23.5% in urban schoolchildren 25. In the present study of acute hospital‐based asthma, the PAF for SPT to any aeroallergen was much higher (57.2%) and most of the effect was explained by mite sensitization.

The higher PAF related to atopy found in our study, particularly in comparison with schoolchildren with wheezing illness from the City of Esmeraldas 25 may be explained by the severity of illness: Children in our study were recruited at a hospital emergency department during an acute asthma attack, while wheezing illness during the previous year was measured by parental questionnaire 18. Atopic asthmatic children tend to have more severe and difficult‐to‐treat disease than non‐atopic asthmatics 26 and may be more likely to require emergency care. Studies from developed countries suggest that atopy, particularly to mite, is a risk factor for hospital admission among asthmatic children 27 and is more common among acute asthmatics compared to asthmatics not requiring hospital re‐admission 28. There are very limited data available from developing countries on the role of atopy as a risk factor for acute asthma. In a study from South Africa, 74.0% of children admitted to a hospital emergency room with acute asthma had a positive SPT to any aeroallergen 29 and is consistent with our own findings of 68% with SPT.

Atopy in our asthmatic population was dominated by mite sensitization: All children with positive sIgE or SPT to any non‐mite aeroallergen were mite sensitized, and the risk of acute asthma was associated with mite IgE titer. A recent study in another more developed tropical urban environment, Singapore, showed that over 70% of 206 individuals sampled from two population‐based cohorts were atopic to mites, that sIgE to other aeroallergens was relatively infrequent (<30%), and that few individuals without mite atopy were sensitized to other aeroallergens 30. It was suggested that mite monosensitization with high‐titer IgE observed in this population contributed to the high prevalence of asthma 30. In our study population, a lower proportion of non‐asthmatic controls than the Singapore study 30 had mite atopy (39%): It is possible that with increasing development, the prevalence of mite atopy may increase translating into a greater prevalence of clinically significant allergic airways disease. The increase in PAF for wheeze/atopy observed previously in urban vs. rural children 7 supports this, and such an effect may be more strongly determined by environmental differences (e.g., potable water and sanitation) than relative affluence measured by monthly income.

The prevalence of rhinovirus infection among the acute asthmatics was high. Previous studies have associated acute respiratory viral infections with acute bronchospasm, particularly rhinovirus infections 31. This association has been described to be stronger among atopic children with high‐titer IgE to mite 32, a trend that was also observed here. The risk of acute asthma may also be linked to high levels of mite exposure in the environment 28. The inflammation induced in the airways in the context of high‐level exposure to mite allergens may modify risk of rhinovirus‐associated wheeze and the development of acute exacerbations.

The role of intestinal helminths in the development of asthma and acute exacerbations remains unclear 13, 26, 33. Allergic sensitization to *Ascaris* has been associated with wheeze and atopy prevalence 34, an effect that may be mediated by a higher degree of atopy among asthmatic children exposed to ascariasis 35, or by a direct effect on airways reactivity by inflammation caused by the host immune response to *Ascaris* larvae migrating through the lungs 36. However, few studies have adjusted these associations for mite specific IgE, given the cross‐reactivity which has been previously described between mite and Ascaris allergens 37. In the present study, in a population of low helminth prevalence, the association between Ascaris IgE and acute asthma disappeared after controlling for mite IgE, indicating confounding by this factor and suggesting that allergic sensitization to Ascaris does not explain acute asthma in our study population.

It has been suggested previously that the hygiene hypothesis may not be valid in Latin America because of the high prevalence of allergy and allergic diseases among the urban poor who live in conditions of poor hygiene 33. Our data indicate that respiratory infections and morbidity, either through a history in early childhood of bronchiolitis or current infections with rhinovirus, are risk factors for acute asthma, while current pet exposures are protective. These observations are consistent with the findings of other studies 4, 38. The presence of Ascaris IgE, which in a population of low prevalence can be used as a marker for exposure 39, was associated with a reduced risk of SPT and is consistent with findings of previous studies 8, 33. Even in Latin America, there is strong evidence that common childhood infections including intestinal worms strongly attenuate atopy but seem to have less effect on asthma symptoms 40. Together, our findings reflect the complexity of the component exposures that make up poor hygiene. Such exposures may affect the risk of acute asthma in different ways – some exposures may protect against asthma, while others may be risk factors. Further, the effect of individual exposures may vary under different circumstances, depending on determinants such as age and intensity of exposure.

Underdiagnosis of asthma (77% of our cases had a previous diagnosis of asthma) likely contributes to poor asthma control – 86% of asthmatics had attended the emergency department the previous year. This may explain the high proportion (45.6% in Ecuador and 73.2% in Latin America 15) of annual costs of asthma‐related health care due to unscheduled healthcare use. None of our asthma cases were receiving long‐term inhaled corticosteroids compared to <6% of asthmatic patients in other regions of Latin America 14, and most relied on emergency care for asthma control.

Our study has several potential limitations. The use of maternal questionnaires to measure risk factors may be subject to recall bias, and we were not able to measure some important potential risk factors in an urban environment such as air pollution. The study had limited power to detect small effects.

In conclusion, acute asthma in a poor urban population in coastal Ecuador was strongly associated with mite atopy, particularly at high titer. Our data highlight the need for further studies in this and similar populations in Latin America to understand better the causes of acute asthma, particularly the role of the interaction between atopy, aeroallergen exposure, and respiratory viral infections that may allow the identification of novel preventive strategies to reduce disease burden.

## Supporting information


**Table S1**. Infant bronchiolitis among acute‐asthmatic patients visiting the emergency room.
**Table S2.** Effect of rhinovirus infection on association between acute asthma and IgE to any mite.

## References

[1] Masoli M , Fabian D , Holt S , Beasley R . Global Initiative for Asthma (GINA) Program. The global burden of asthma: executive summary of the GINA Dissemination Committee report. Allergy 2004: 59: 469–78. 15080825 10.1111/j.1398-9995.2004.00526.x

[2] Bousquet J , Dahl R , Khaltaev N . Global alliance against chronic respiratory diseases. Eur Respir J 2007: 29: 233–9. 17264322 10.1183/09031936.00138606

[3] Pearce N , Ait‐Khaled N , Beasley R , et al. Worldwide trends in the prevalence of asthma symptoms: phase III of the International Study of Asthma and Allergies in Childhood (ISAAC). Thorax 2007: 62: 758–66. 17504817 10.1136/thx.2006.070169 PMC2117323

[4] Barreto ML , Cunha SS , Fiaccone R , et al. Poverty, dirt, infections and non‐atopic wheezing in children from a Brazilian urban center. Respir Res 2010: 11: 167. 21122116 10.1186/1465-9921-11-167 PMC3002921

[5] Cooper PJ . Interactions between helminth parasites and allergy. Curr Opin Allergy Clin Immunol 2009: 9: 29–37. 19106698 10.1097/ACI.0b013e32831f44a6 PMC2680069

[6] Fischer GB , Camargos PA , Mocelin HT . The burden of asthma in children: a Latin American perspective. Paediatr Respir Rev 2005: 6: 8–13. 15698808 10.1016/j.prrv.2004.11.002

[7] Cooper PJ , Vaca M , Rodriguez A , et al. Hygiene, atopy and wheeze‐eczema‐rhinitis symptoms in schoolchildren from urban and rural Ecuador. Thorax 2014: 69: 232–9. 24105783 10.1136/thoraxjnl-2013-203818 PMC3932750

[8] Cooper PJ , Chico ME , Bland M , Griffin GE , Nutman TB . Allergic symptoms, atopy, and geohelminth infections in a rural area of Ecuador. Am J Respir Crit Care Med 2003: 168: 313–7. 12714349 10.1164/rccm.200211-1320OC

[9] Weinmayr G , Weiland SK , Bjorksten B , et al. Atopic sensitization and the international variation of asthma symptom prevalence in children. Am J Respir Crit Care Med 2007: 176: 565–74. 17575099 10.1164/rccm.200607-994OC

[10] Eder W , Ege MJ , von Mutius E . The asthma epidemic. N Engl J Med 2006: 355: 2226–35. 17124020 10.1056/NEJMra054308

[11] Genuneit J . Exposure to farming environments in childhood and asthma and wheeze in rural populations: a systematic review with meta‐analysis. Pediatr Allergy Immunol 2012: 23: 509–18. 22625206 10.1111/j.1399-3038.2012.01312.x

[12] Douwes J , Pearce N . Commentary: The end of the hygiene hypothesis? Int J Epidemiol 2008: 37: 570–2. 18456712 10.1093/ije/dyn077

[13] Cooper PJ , Rodrigues LC , Barreto ML . Influence of poverty and infection on asthma in Latin America. Curr Opin Allergy Clin Immunol 2012: 12: 171–8. 22391754 10.1097/ACI.0b013e3283510967 PMC7612855

[14] Neffen H , Fritscher C , Schacht FC , et al. Asthma control in Latin America: the Asthma Insights and Reality in Latin America (AIRLA) survey. Rev Panam Salud Publica 2005: 17: 191–7. 15826399 10.1590/s1020-49892005000300007

[15] Neffen H , Gonzalez SN , Fritscher CC , Dovali C , Williams AE . The burden of unscheduled health care for asthma in Latin America. J Investig Allergol Clin Immunol 2010: 20: 596–601. 21314001

[16] Dougherty RH , Fahy JV . Acute exacerbations of asthma: epidemiology, biology and the exacerbation‐prone phenotype. Clin Exp Allergy 2009: 39: 193–202. 19187331 10.1111/j.1365-2222.2008.03157.x PMC2730743

[17] Reddel HK , Taylor DR , Bateman ED , et al. An official American Thoracic Society/European Respiratory Society statement: asthma control and exacerbations: standardizing endpoints for clinical asthma trials and clinical practice. Am J Respir Crit Care Med 2009 Jul 1: 180: 59–99. 19535666 10.1164/rccm.200801-060ST

[18] ISAAC Phase Two Study Group . ISSAC Phase Two Study Modules. Available at: http://isaac.auckland.ac.nz/phases/phasetwo/phasetwomodules.pdf. Accessed February, 2013.

[19] Cooper PJ , Chico ME , Vaca MG , et al. Risk factors for asthma and allergy associated with urban migration: background and methodology of a cross‐sectional study in Afro‐Ecuadorian school children in Northeastern Ecuador (Esmeraldas‐SCAALA Study). BMC Pulm Med 2006: 13: 24. 16970809 10.1186/1471-2466-6-24 PMC1578586

[20] Ralston SL , Lieberthal AS , Meissner HC , et al. Clinical practice guideline: the diagnosis, management, and prevention of bronchiolitis. Pediatrics 2014: 134: e1474–502. 25349312 10.1542/peds.2014-2742

[21] World Health Organization . Parasitic Diseases Programme. Diagnostic techniques for intestinal parasitic infections (IPI) applicable to primary health care (PHC) services. 1985.

[22] Rosa MJ , Divjan A , Hoepner L , et al. Fractional exhaled nitric oxide exchange parameters among 9‐year‐old inner‐city children. Pediatr Pulmonol 2011: 46: 83–91. 20848585 10.1002/ppul.21328 PMC3056274

[23] Hogman M , Drca N , Ehrstedt C , Merilainen P . Exhaled nitric oxide partitioned into alveolar, lower airways and nasal contributions. Respir Med 2000: 94: 985–91. 11059953 10.1053/rmed.2000.0872

[24] Budge PJ , Griffin MR , Edwards KM , et al. A household‐based study of acute viral respiratory illnesses in Andean children. Pediatr Infect Dis J 2014: 33: 443–7. 24378948 10.1097/INF.0000000000000135 PMC4223552

[25] Endara P , Vaca M , Platts‐Mills T , et al. Effect of urban versus rural residence on the association between atopy and wheeze in Latin America: findings from a case‐control analysis. Clin Exp Allergy 2015: 45: 438–47. 25200287 10.1111/cea.12399 PMC4413357

[26] Pereira MU , Sly PD , Pitrez PM , et al. Nonatopic asthma is associated with helminth infections and bronchiolitis in poor children. Eur Respir J 2007: 29: 1154–60. 17331964 10.1183/09031936.00127606

[27] Rasmussen F , Taylor DR , Flannery EM , et al. Risk factors for hospital admission for asthma from childhood to young adulthood: a longitudinal population study. J Allergy Clin Immunol 2002: 110: 220–7. 12170261 10.1067/mai.2002.125295

[28] Murray CS , Poletti G , Kebadze T , et al. Study of modifiable risk factors for asthma exacerbations: virus infection and allergen exposure increase the risk of asthma hospital admissions in children. Thorax 2006: 61: 376–82. 16384881 10.1136/thx.2005.042523 PMC2111190

[29] Kling S , Donninger H , Williams Z , et al. Persistence of rhinovirus RNA after asthma exacerbation in children. Clin Exp Allergy 2005: 35: 672–8. 15898992 10.1111/j.1365-2222.2005.02244.x

[30] Andiappan AK , Puan KJ , Lee B , et al. Allergic airway diseases in a tropical urban environment are driven by dominant mono‐specific sensitization against house dust mites. Allergy 2014 Apr: 69: 501–9. 24456108 10.1111/all.12364 PMC4240470

[31] Heymann PW , Platts‐Mills T , Johnston SL . Role of viral infections, atopy and antiviral immunity in the etiology of wheezing exacerbations among children and young adults. Pediatr Infect Dis J 2005: 24: S217–22. 16378049 10.1097/01.inf.0000188164.33856.f9

[32] Soto‐Quiros M , Avila L , Odio S , et al. High titers of IgE antibody to dust mite allergen and risk for wheezing among asthmatic children infected with rhinovirus. J Allergy Clin Immunol 2012: 129: 1499–505. 22560151 10.1016/j.jaci.2012.03.040 PMC3792652

[33] Moncayo AL , Vaca M , Oviedo G , et al. Risk factors for atopic and non‐atopic asthma in a rural area of Ecuador. Thorax 2010 May: 65: 409–16. 20435862 10.1136/thx.2009.126490 PMC2988616

[34] Moncayo AL , Vaca M , Oviedo G , et al. Effects of geohelminth infection and age on the associations between allergen‐specific IgE, skin test reactivity and wheeze: a case‐control study. Clin Exp Allergy 2013: 43: 60–72. 23278881 10.1111/cea.12040 PMC3563216

[35] Hunninghake GM , Soto‐Quiros ME , Avila L , et al. Sensitization to *Ascaris lumbricoides* and severity of childhood asthma in Costa Rica. J Allergy Clin Immunol 2007: 119: 654–61. 17336615 10.1016/j.jaci.2006.12.609

[36] Cooper PJ , Rodrigues LC , Cruz AA , Barreto ML . Asthma in Latin America: a public heath challenge and research opportunity. Allergy 2009 Jan: 64: 5–17. 19076533 10.1111/j.1398-9995.2008.01902.x

[37] Acevedo N , Caraballo L . IgE cross‐reactivity between *Ascaris lumbricoides* and mite allergens: possible influences on allergic sensitization and asthma. Parasite Immunol 2011: 33: 309–21. 21388422 10.1111/j.1365-3024.2011.01288.x

[38] Takkouche B , Gonzalez‐Barcala FJ , Etminan M , Fitzgerald M . Exposure to furry pets and the risk of asthma and allergic rhinitis: a meta‐analysis. Allergy 2008: 63: 857–64. 18588551 10.1111/j.1398-9995.2008.01732.x

[39] Fincham JE , Markus MB , van der Merwe L , Adams VJ , van Stuijvenberg ME , Dhansay MA . Ascaris, co‐infection and allergy: the importance of analysis based on immunological variables rather than egg excretion. Trans R Soc Trop Med Hyg 2007: 101: 680–2. 17254621 10.1016/j.trstmh.2006.11.006

[40] Alcantara‐Neves NM , Veiga RV , Dattoli VC , et al. The effect of single and multiple infections on atopy and wheezing in children. J Allergy Clin Immunol 2012: 129: 359–67, 367.e1–3. 22035877 10.1016/j.jaci.2011.09.015 PMC5015705

