# Supplementary material for: Risk factors for acute asthma in tropical America: a case–control study in the City of Esmeraldas, Ecuador
Source: Pediatr Allergy Immunol. 2015 Jul 27;26(5):423–30. doi: 10.1111/pai.12401 (PMC4737128; doi:10.1111/pai.12401)
Supplement: Supplementary file 1 — Table S1. Infant bronchiolitis among acute‐asthmatic patients visiting the emergency room. Table S2. Effect of rhinovirus infection on association between acute asthma and IgE to any mite. [file PAI-26-423-s001.docx]

Supplementary Table S1: Infant bronchiolitis among acute-asthmatic patients visiting the emergency room.

| **Sex** | **Age** | **History of bronchiolitis^&^** | **No history of bronchiolitis** | **P value*** |
| --- | --- | --- | --- | --- |
| Male | 5-9 years | 12 (63%) | 7 (37%) | 1.00 |
|  | 10-15 years | 8 (67%) | 4 (33%) |  |
| Female | 5-9 years | 12 (75%) | 4 (25%) | 0.143 |
|  | 10-15 years | 6 (46%) | 7 (54%) |  |

* P values were calculated using Fisher’s Exact Test.

&: History of bronchiolitis was defined as a positive answer to the question: ‘Did your child suffer an episode of wheezing and difficulty breathing during the first 2 years ol life?’.

Supplementary Table S2. Effect of rhinovirus infection on association between acute asthma and IgE to any mite.

| Allergen IgE titer | Rhinovirus Positive (37) | | | | Rhinovirus Negative (121) | | | |
| --- | --- | --- | --- | --- | --- | --- | --- | --- |
|  | Cases  N (%) | Controls  N (%) | OR (95%CI) | P value | Cases  N (%) | Controls  N (%) | OR (95%CI) | P value |
| Any mite |  |  |  |  |  |  |  |  |
| <=0.70 | 4 (17) | 7 (54) | 1 |  | 7 (20) | 57 (66) | 1 |  |
| >0.70 | 20 (83) | 6 (46) | 65 (0,0-5658381) | 0,471 | 28 (80) | 29 (34) | 6,2 (2,1-18,3) | 0,001 |
| Ascaris |  |  |  |  |  |  |  |  |
| <=0.70 | 7 (29) | 11 (85) | 1 |  | 18 (51) | 57 (66) | 1 |  |
| >0.70 | 17 (71) | 2 (15) | 65 (0,0-702391) | 0,378 | 17 (49) | 29 (34) | 1,79 (0,8-4,1) | 0,168 |

OR: Odds Ratio; CI: Confidence Interval
